# Editorial: The functions of extracellular vesicles in melanoma

**DOI:** 10.3389/fcell.2023.1232182

**Published:** 2023-06-14

**Authors:** Susana García-Silva, Miriam Galvonas Jasiulionis, Alberto Benito-Martín

**Affiliations:** ^1^ Microenvironment and Metastasis Laboratory, Molecular Oncology Programme, Spanish National Cancer Research Center (CNIO), Madrid, Spain; ^2^ Departamento de Farmacologia, Escola Paulista de Medicina, Universidade Federal de São Paulo, São Paulo, Brazil; ^3^ Facultad de Medicina, Unidad de Investigación Biomédica, Universidad Alfonso X El Sabio (UAX), Madrid, Spain

**Keywords:** melanoma, extracellular vescicles, biomarkers, microRNAs biomarkers, metastasis

Melanoma, the most aggressive form of skin cancer, has shown a rising incidence worldwide in recent years. Despite advancements in treatment options, the understanding of the complex mechanisms underlying melanoma progression and metastasis remains a significant challenge (Atkins et al., 2021). Recently, extracellular vesicles (EVs) have emerged as key players in intercellular communication (van Niel et al., 2022), offering new insights into melanoma biology and potential implications for diagnosis, prognosis, and therapeutic strategies. In this Research Topic of Frontiers in Cell and Developmental Biology, we have curated a Research Topic of articles covering different aspects of EVs biology in melanoma.

The study of melanoma-derived vesicles has provided very relevant contributions to the knowledge of the functional roles of EVs in tumor progression and in particular, regarding the formation of pre-metastatic niches and metastatic colonization (Schnaeker et al., 2004; Chen et al., 2006; Peinado et al., 2012; Chen et al., 2018; García-Silva et al., 2021). For adding more evidence to the roles of EVs in metastatic colonization, Chen et al. show in this Research Topic how high-metastatic melanoma cells can augment the proliferation and colonization capability of low-metastatic melanoma cells by transferring exosomal miR-411-5p, thus providing a mechanism for EV-meditated communication between tumor clones. This publication has generated a commentary (Li et al., 2022) inspired by this enhancement of metastatic “fitness” due to surrounding tumor EV uptake.

On the other hand, the promising use of EVs as biomarkers continues to gather most of the interest of the scientific community. We had the pleasure to include here additional works addressing EV applicability to melanoma diagnosis and therapy monitoring. Gerloff et al. profile miRNAs derived from human melanocyte and melanoma cells and their secreted EVs. Remarkably, they observe that although miRNA content greatly differ between melanocytes and melanoma cells, there is a substantial similarity in miRNA cargo between EVs from melanocytes and melanoma cells. They also identify six miRNAs enriched in both melanoma cells and melanoma cell-derived EVs. Among them, miR-92b-3p, miR-182-5p and miR-183-5p were also significantly augmented in EVs derived from serum of melanoma patients. Their findings support the hypothesis that miRNAs derived from EVs can serve as prognostic or diagnostic markers for liquid biopsy in melanoma. Following a similar approach but in uveal melanoma, Wróblewska et al. identify hsa-miR-144-5p, hsa-miR-191-5p, hsa-miR-223-3p, hsa-miR -483-5p and hsa-miR-203a as potential candidates for early detection of this ocular tumor type.

Crescitelli et al. analyze the presence of tumor-specific DNA mutations in EVs directly isolated from human melanoma fresh metastatic biopsies and compared the results with tumor tissue DNA and plasma-derived DNA. A panel of 34 melanoma-related genes was investigated using ultra-sensitive sequencing (SiMSen-seq). Interestingly, they found that the mutant allele frequency was higher in DNA isolated from tumor-derived EVs compared to total DNA directly extracted from plasma DNA. These findings support the idea of a potential role for tumor EVs as a valuable source of mutational information in melanoma.

The use of biological vesicles as nanocarriers is also a growing field that could offer some advantages in comparison to other chemically designed formulations (Klyachko et al., 2020; Hou et al., 2022). Concerning the drug delivery systems, Bhattacharya et al. investigate the encapsulation of Dacarbazine in lipid nanoparticles and compare it to free delivery of this drug and study its effects in melanoma-bearing rats.

Lopez-Borrego et al. contribution to this topic is focused on the interplay between therapy resistance and EV surface cargo. They show that BRAF and MAPK inhibitors alone or in combination modulate NKG2D-ligands in melanoma cells and secreted EVs, together with other NK activating ligands *in vitro*. NKG2D-ligand fluctuation during MAPKi treatment could have different consequences for the immune response against the tumor and could even contribute to therapy resistance. The authors additionally pioneered the testing of several NKG2D-ligands and other melanoma antigens in the sera of metastatic patients under targeted therapy, both as soluble and vesicle-released proteins as a way to envision patient immune competence, which has proven to be critical to improve survival rates.

Finally, we have included two reviews focusing in several aspects of EVs role in melanoma. In a particularly interesting mini-review, Lothar C. Dieterich discusses the mechanistic insights of EV-mediated regulation of various immune cell types, including the effects on inflammatory, apoptotic, stress-sensing, and immune checkpoint pathways as well as antigen-dependent responses. He also highlights the current challenges that need to be overcome to determine the clinical relevance of these various mechanisms and to develop corresponding therapeutic approaches to promote tumor immunity and immunotherapy responsiveness in melanoma patients in the future. And last but not least, the editorial team of this Research Topic Benito-Martín et al. provides an in-depth overview of the characteristics of melanoma-derived EVs and their role in melanoma progression highlighting key advances and remaining open questions in the field. We have focused in melanoma EVs heterogeneity, the complexities associated with vesicle’s cargo, the role of EVs in normal skin physiology and melanoma progression. We try to offer a fresh update on the vesicles-mediated interplay between melanoma cells and the microenvironment and the formation of pre-metastatic niches in the lymph nodes and distant organs.

Although this Research Topic collection offers new insights on the role of EVs in melanoma, open questions and challenges still remain ahead. The complex network of effects between tumor, immune and stromal-derived small EVs is far from being understood, like the influence of EVs in cell adhesion and ECM degradation, all of them key features in metastatic dissemination. Extracellular vesicles heterogeneity, a novel path recently opened, is changing the way we understand the interactions in the microenvironment, and it is gradually reshaping many cancer research assumptions (Wang et al., 2023). It is possible that melanoma would be modulated differently depending on the cell type and the physiological/pathological status.

In addition, the role of EVs in the systemic effects induced during the terminal stages of melanoma remains unexplored. Considering the abundant pro-coagulant cargo present in small EVs (Broggi et al., 2019; García-Silva et al., 2019), the systemic pro-thrombotic risk of late stage patients could be at least partially, mediated by tumor EVs. Even more, the role of extracellular vesicles as disease biomarkers is far from being fully developed and rigorous approaches showing clinical relevance are still awaited.

We hope that this Research Topic of articles would capture the interest of the EV community and provide inspiration for future research works addressing the current open questions in the field.
